# Divergence of rhizosphere microbial communities between females and males of the dioecious *Hippophae tibetana* at different habitats

**DOI:** 10.1128/spectrum.01670-24

**Published:** 2024-09-11

**Authors:** YiFan Mao, Ni Li, YaLi Huang, DaWei Chen, Kun Sun

**Affiliations:** 1 College of Life Sciences, Northwest Normal University, Lanzhou, Gansu, China; Ocean University of China, Qingdao, China

**Keywords:** dioecious plant, rhizosphere microbe community, different habitats, *Hippophae tibetana*

## Abstract

Females and males of dioecious plants have evolved sex-specific characteristics in terms of their morphological and physiological properties. However, little is known about the difference in rhizosphere microbes in dioecious plants. In this study, we used amplicon sequencing to analyze the differences in rhizosphere microbial diversity and community composition of males and females of dioecious *Hippophae tibetana* at different habitats, and their key factors in driving the differences were investigated. The results showed that there were differences in the diversity, community composition, and connectivity and complexity of the co-occurrence network of rhizosphere microbes between females and males of the dioecious *H. tibetana* at different habitats. Zoopagomycota is a unique phylum of rhizosphere fungi in the males of the dioecious *H. tibetana*, while Dependentiae is a unique phylum of rhizosphere bacteria in the females of the dioecious *H. tibetana*. Linear discriminant analysis effect size (LEfSe) analysis indicated significant enrichment of species at different levels, suggesting that these species could be potential biomarkers for females and males of *H. tibetana*. Spearman’s analysis showed that the dominant genera of rhizosphere fungi were significantly positively correlated with soil physicochemical properties (total nitrogen and phosphorus; organic matter; available phosphorus, potassium and nitrogen; salt content; water content). PICRUSt and FUNGuild predictive analysis indicated that the function of rhizosphere fungi was different between females and males of the dioecious *H. tibetana* at different habitats, while metabolites were the dominant functions of rhizosphere bacteria in all samples. These results highlighted the sexual discrimination of rhizosphere microbes on the dioecious plants and provided important knowledge for females and males of the dioecious plant–microbe interaction.

**IMPORTANCE:**

This study explores the differences in rhizosphere microbes of dioecious *Hippophae tibetana* at different habitats and their key factors in driving the differences. Through employing amplicon sequencing techniques, we found that rhizosphere microbial communities and diversity were different between females and males of the dioecious *H. tibetana* at different habitats, and there notably existed unique phylum and potential biomarkers of rhizosphere microbes between females and males of the dioecious *H. tibetana*. Rhizosphere fungi were significantly positively correlated with soil physicochemical properties. This study reveals the differences in rhizosphere microbes of dioecious *H. tibetana* at different habitats and driving factors; it also contributes to our understanding of the dioecious plant–microbe interaction.

## INTRODUCTION

Dioecious plant species are widely distributed among different phyla ranging from bryophytes and gymnosperms to angiosperms and are an important component of terrestrial ecosystems (1), which have evolved independently from hermaphroditic ancestors, including 15,600 species in 987 genera and 175 families (2). Previous studies have reported that male and female plants show sexual dimorphism in growth, defense, and storage (3). Due to the greater reproductive effort compared with males (4), female plants were found to require more favorable habitats to survive (5), whereas male plants have a higher resistance or tolerance to various stressful environments compared with female plants (6). Yu *et al*. (7) found that male-biased *Populus euphratica* populations indicate a superior adaptation compared with female plants under relatively dry and infertile soils condition (nutrient-poor environments). Jiang *et al*. (8) reported that male *P. cathayana* have a greater tolerance to enhanced UV-B radiation than females. Recently, the differences between female and male plants in the morphology, resource habitat and reproductive habitat, stress adaptive capacity, and nutrient availability in stressful environments have become a topic of considerable interest (9).

During plant evolution, plant microbiota or microbiome have coevolved over millions of years with their host and are linked to plant fitness as a “holobiont” (10). They establish complex interactions with plants and provide a number of life-supporting functions for their host—such as promoting nutrient uptake, enhancing resistance to abiotic stresses, and preventing diseases (11). These plant microbiota or microbiome inhabit the plant surface (e.g., Phyllosphere microbes), inside plant tissues (endophyte), and are acquired from the environment (e.g., rhizosphere microbes) (12). Among them, rhizosphere microbes played an important role in host nutrient utilization, stress tolerance, plant health, and adaptation (13). The increasing interest in the microbial community in the rhizosphere of plants is driven by the potential of fungi and bacteria to modulate stress tolerance by elevating defensive responses or competition for substrates in hosts (14). Therefore, analysis of the diversity and composition of rhizosphere microbes could provide valuable information for understanding the ecological and evolutionary factors that affect the population structure of dioecious plants. However, the rhizosphere microbial community and diversity varies with different habitats, which is still unknown.

*Hippophae tibetana* is a perennial shrub with dioecious and parthenogenetic clonal reproduction and is a pioneer species in community succession with important ecological and economic value (15). It is distributed across the Tibetan Plateau and Himalayan habitat at altitudes of 2,800–5,200 m (15). Previous studies have focused on the differences in morphology, physiology, and adaptation strategies of female and male *H. tibetana* (15). However, the underlying ecological processes in regulating plant–microbiome–environment interactions are still unknown. Meanwhile, sex-specific differences of the composition of rhizosphere microbes between females and males of the dioecious *H. tibetana* are not clear. Therefore, in this study, we collected the male and female plants of *H. tibetana* from different habitats to analyze the differences in rhizosphere microbial diversity and community composition, and their key environmetal factors in driving the differences were investigated. Our results provided important knowledge for females and males of the dioecious plant–microbe interaction.

## MATERIALS AND METHODS

### Experimental materials

In September 2023, the soil samples were collected from two habitats at Tianzhu County, Wuwei City, Gansu Province, China (102°45′11.26″E, 37°12′47.50″N, 2,903.56 m. The average annual precipitation is 410.50 mm. The average annual potential evaporation is 1,592 mm, which is 3.8 times the annual precipitation. The average annual temperature is −0.1°C. The total annual sunshine hours is 2,600 hours). Two habitats were kept 5 km apart; habitat A was located beside the beach (Fig. S1a), and habitat B was located in the meadow (Fig. S1b). Rhizosphere soil physicochemical properties of the two habitats are shown in Table 1. Three biological replicates were collected for each category. The samples were stored in an ice box and transported immediately to the laboratory (within 3 hours). The root system of the male and female *H. tibetana* plants was shaken to shake off the soil not closely attached to the root system, and the rhizosphere soil closely attached to the root system was collected as the rhizosphere soil. As for the root surface soil, visible soil was removed, and the roots were suspended in sterile distilled water. The mixture was subjected to ultrasonic cleaning (KQ5200DE, Shumei Ultrasonic Instrument Co., LTD, Kunshan city, China) for 10  minutes (200 W, 30/30 s). The final soil solution was centrifuged at 12,000  r/min for 10  minutes to remove the supernatant, leaving behind the tightly adhered root surface soil (16). The samples were kept at −80°C until the extraction of DNA.

**TABLE 1:** Soil physicochemical properties of two habitats^*a*^

Sample	TN	TP	TK	QP	QN	QK	OM	SW	SC	pH
AFX	4.61 ± 0.10b	670.16 ± 8.20 c	17.62 ± 0.17 a	2.71 ± 0.50b	249.08 ± 21.14b	288.42 ± 7.82 c	83.22 ± 2.97b	0.71 ± 0.01 a	0.039 ± 0.00b	6.86 ± 0.02 a
AMX	4.52 ± 0.00b	701.46 ± 5.84b	15.05 ± 0.17b	3.11 ± 0.13b	250.99 ± 4.06b	256.91 ± 4.66d	84.19 ± 1.41b	0.71 ± 0.01 a	0.04 ± 0.01b	6.87 ± 0.01 a
BFX	10.23 ± 0.11 a	848.28 ± 7.07 a	13.83 ± 0.23 c	5.07 ± 0.60 a	606.81 ± 22.80 a	505.66 ± 11.68 a	208.38 ± 0.55 a	0.58 ± 0.12 a	0.068 ± 0.00 a	6.77 ± 0.30b
BMX	10.03 ± 0.84 a	860.67 ± 3.16 a	11.72 ± 0.64d	5.10 ± 0.13 a	618.05 ± 8.20 a	472.48 ± 3.50b	207.93 ± 0.84 a	0.58 ± 0.12 a	0.069 ± 0.00 a	6.76 ± 0.23b

^*a*^ Note: TN represents total nitrogen, TP represents total phosphorus, TK represents total potassium, QP represents available phosphorus, QN represents available nitrogen, QK represents available potassium, OM represents organic matter, SW represents water content, and SC represents salt content of soil. Different letters above the bars indicate the differences are significant at *P* < 0.05.

### DNA extraction, polymerase chain reaction (PCR) amplifcation, and sequence processing

The total genomic DNA was extracted from all samples by using the MOBIO Power -Soil Kit (MOBIO Laboratories, Inc., Carlsbad, CA, USA), according to the manufacturer’s instructions. The DNA extracts were analyzed for their concentration using the NanoDrop spectrophotometer (Thermo Fisher Scientific, Model 2000, Waltham, MA, USA) and stored at −20°C for 2 days (in Tris-EDTA) and then underwent PCR amplification. The primers 341F (5′-CCTACGGGNGGCWGCAG-3′) and 805R (5′-GACTACHVGGGTATCTAATCC-3′) were used to amplify the bacterial 16S rRNA gene. The amplification of fungal ITS genes was performed using the primers ITS1F (5′-CTTGGTCATTTAGAGGAAGTAA-3′) and ITS2R (5′-GCTGCGTTCTTCATCGATGC-3′). The PCR products were examined using agarose gel electrophoresis. Successful PCR products of all samples were pooled and purified using the EasyPureTM PCR Cleanup/Gel Extraction Kit (Axygen Biosciences, Union City, CA, USA) according to the manufacturer’s instructions. Purified PCR products were sequenced on an Illumina NovaSeq platform (Novogene Co., Ltd., Beijing, China).

### Data analysis

The fungal ITS sequences and bacterial 16S rRNA genes were analyzed using QIIME 2 (17). Fungal and bacterial sequences were trimmed and assigned to each sample based on their barcodes. The UPARSE-OTUref was used to classify operational taxonomic units (OTUs) at the species level by searching all sequences against the Silva bacterial 16S database (18). OTUs were classified at the species level by searching against the UNITE fungal database (19). Sequences were binned into operational taxonomic units at 97% similarity level by using USEARCH software (http://drive5.com/uparse/) (18). Rarefaction analysis based on Mothur v.1.21.1 was conducted to reveal the diversity indices, including goods coverage, Chao 1, and Shannon (20). Nonmetric multidimensional permutation analysis (NMDS) was used to analyze the discrepancies between the samples at the level of OTUs based on the Bray–Curtis distance (9). Linear discriminant analysis effect size (LEfSe) was used for biomarker analysis to explore the dissimilarities in the species composition among the samples (21). Co-occurrence network analyses were carried out using the “igraph” package in R, with networks visualized through Gephi (22). Correlation analysis between rhizosphere microbe and soil physicochemical properties was done using the Spearman method (23). Ecological functions were annotated by PICRUSt for the 16S rDNA OTU and FUNGuild v1.0 for the ITS OUT (24). Soil physicochemical properties and Shannon and Chao1 indices were analyzed by using SPSS 17.0 (SPSS Inc., IL, USA) software for variance (one-way ANOVA) and Duncan’s multiple range test (*P* < 0.05).

## RESULTS

### Analysis of alpha diversity

A total of 393,015 and 308,177 effective tags were obtained for the fungal and bacterial samples, with the library coverage of the samples being higher than 0.997, which indicates that the sequencing data confidently reflected the structure of the endophytic fungi and bacterial community of the samples (Table S1).

**Fig 1:**
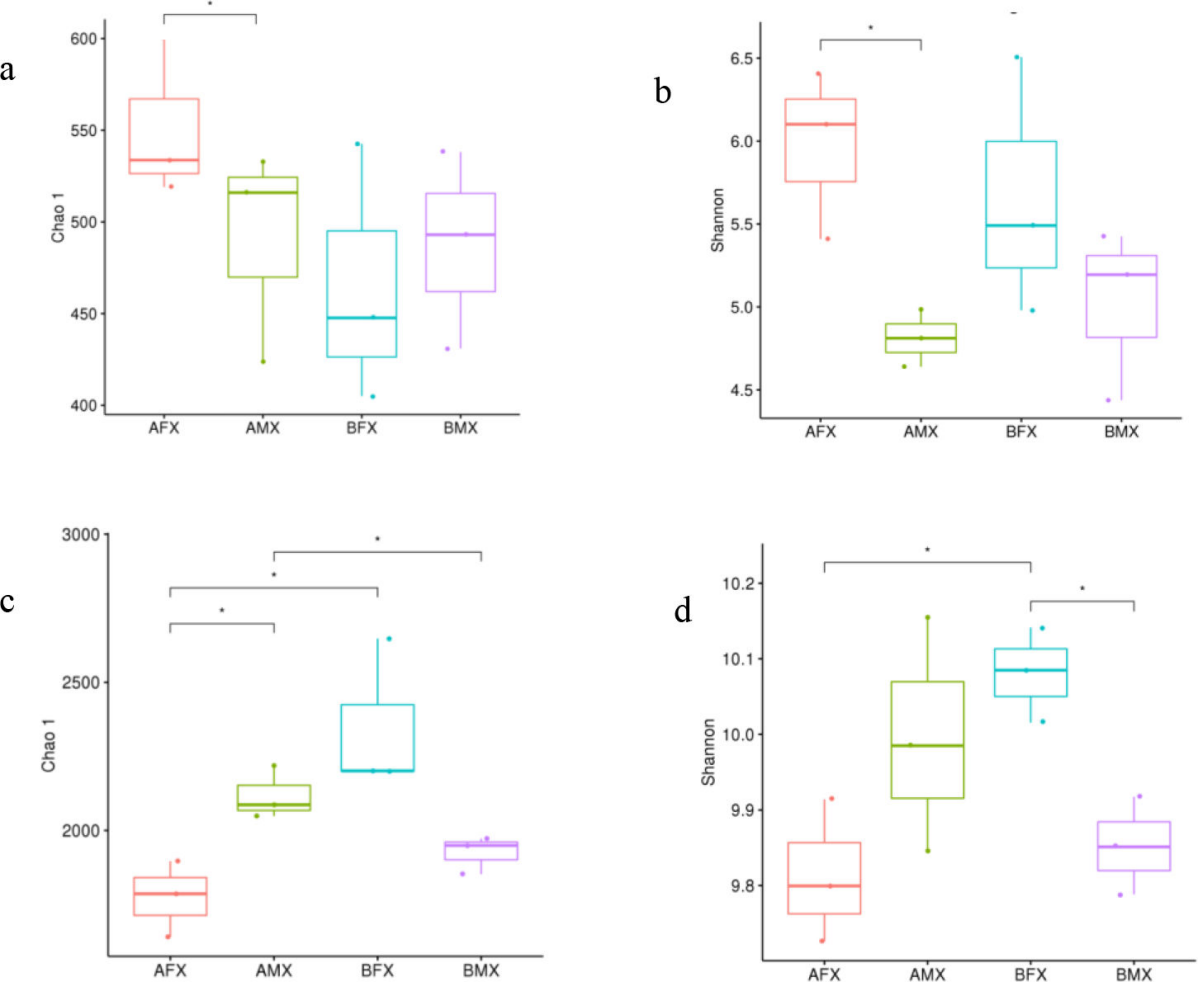
Alpha diversity of rhizosphere fungi and bacteria of females and males of the dioecious *H. tibetana* at different habitats. Note: Chao 1 index of rhizosphere fungi (**a**), Shannon index of rhizosphere fungi (**b**), Chao 1 index of rhizosphere bacteria (**c**), Shannon index of rhizosphere bacteria (**d**). “*” represents that the differences are significant at *P* < 0.05 (*t*-test)

The alpha diversity analysis (Chao1 and Shannon indices) showed differences among females and males of the dioecious *H. tibetana* at different habitats. In the females and males of the dioecious *H. tibetana* at habitat A, the rhizosphere fungi’ chao1 and Shannon indices of the AFX sample were 60.23 and 1.16 higher than those of the AMX sample, respectively (*P* < 0.05) (Fig. 1a and b), whereas rhizosphere bacteria’ chao1 and Shannon indices of the AFX sample were 343.64 and 0,19 lower than those of the AMX sample, respectively (Fig. 1c and d). In the females and males of the dioecious *H. tibetana* at habitat B, rhizosphere fungi’ chao1 index of the BFX sample was 22.28 lower than that of the BMX sample (Fig. 1a), rhizosphere fungi’ Shannon index of the BFX sample was 0.64 higher than that of the BMX sample (Fig. 1b), while rhizosphere bacteria chao1 and shannon indices of the BFX sample were 424.31 and 0.23 higher than those of the BMX sample, respectively (*P* < 0.05) (Fig. 1c and d).

### Community composition

**Fig 2:**
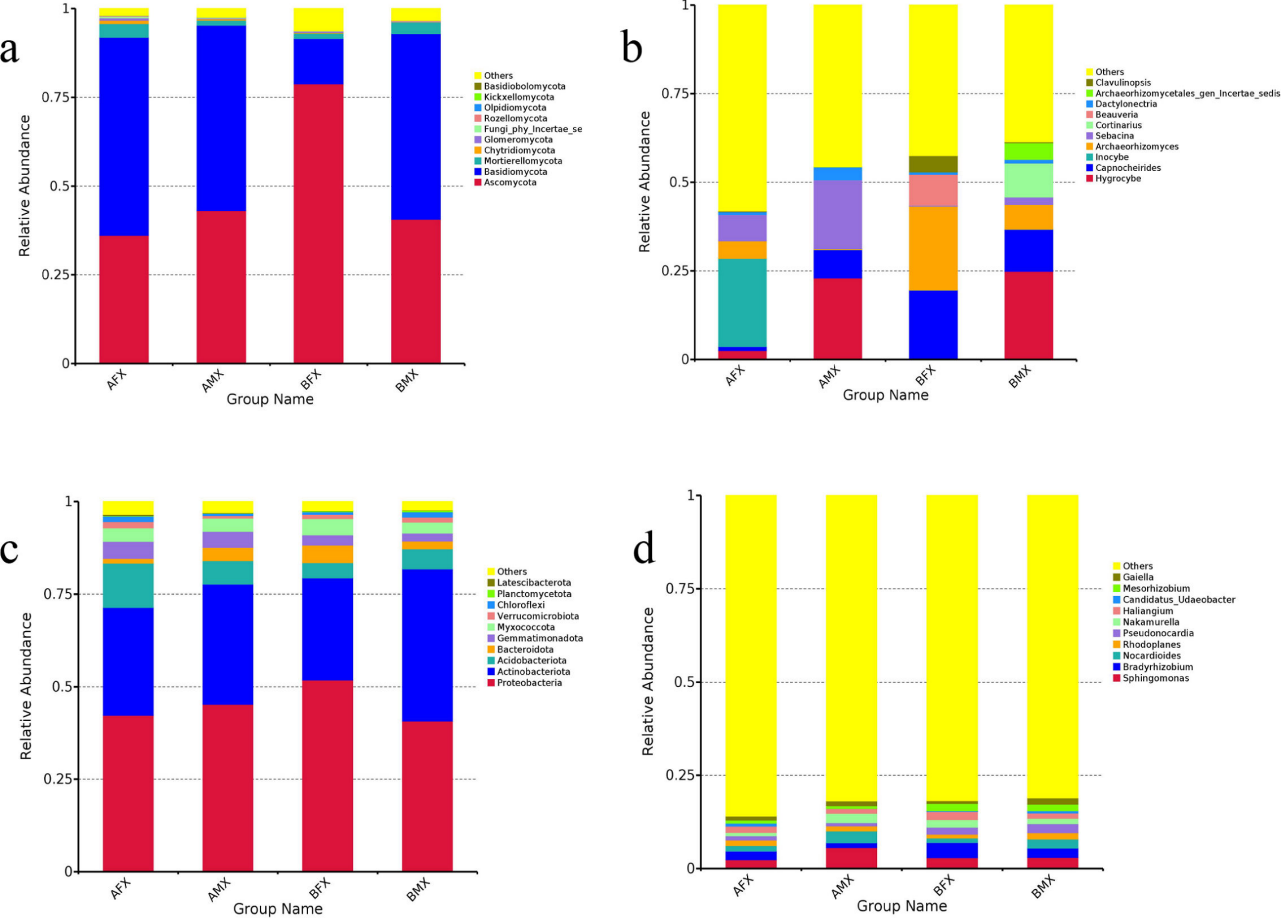
Relative abundances of the rhizosphere microbes between females and males of the dioecious *H. tibetana* at different habitats. Note: rhizosphere fungi at the phylum level (**a**), rhizosphere fungi at the genus level (**b**), rhizosphere bacteria at the phylum level (**c**), and rhizosphere bacteria at the genus level (**d**). “Other” represents the total relative abundance outside the top ten maximum relative abundance levels.

The fungal OTUs were assigned to 15 phyla and 436 genera in all samples. At the phylum level, Basidiomycota was the dominant fungal phylum in the AFX, AMX, and BMX samples, with relative abundances of 52.21% and 55.71%, while Ascomycota was the dominant fungal phylum in the BFX sample (78.82%) (Fig. 2a). At the genus level, *Inocybe* was the dominant fungal genus in the AFX sample (24.82%), *Hygrocybe* was the dominant fungal genus in the AMX and BMX samples (22.97% and 24.88%, respectively), and *Archaeorhizomyces* was the dominant fungal genus in the BFX sample (23.65%) (Fig. 2b).

**Fig 3:**
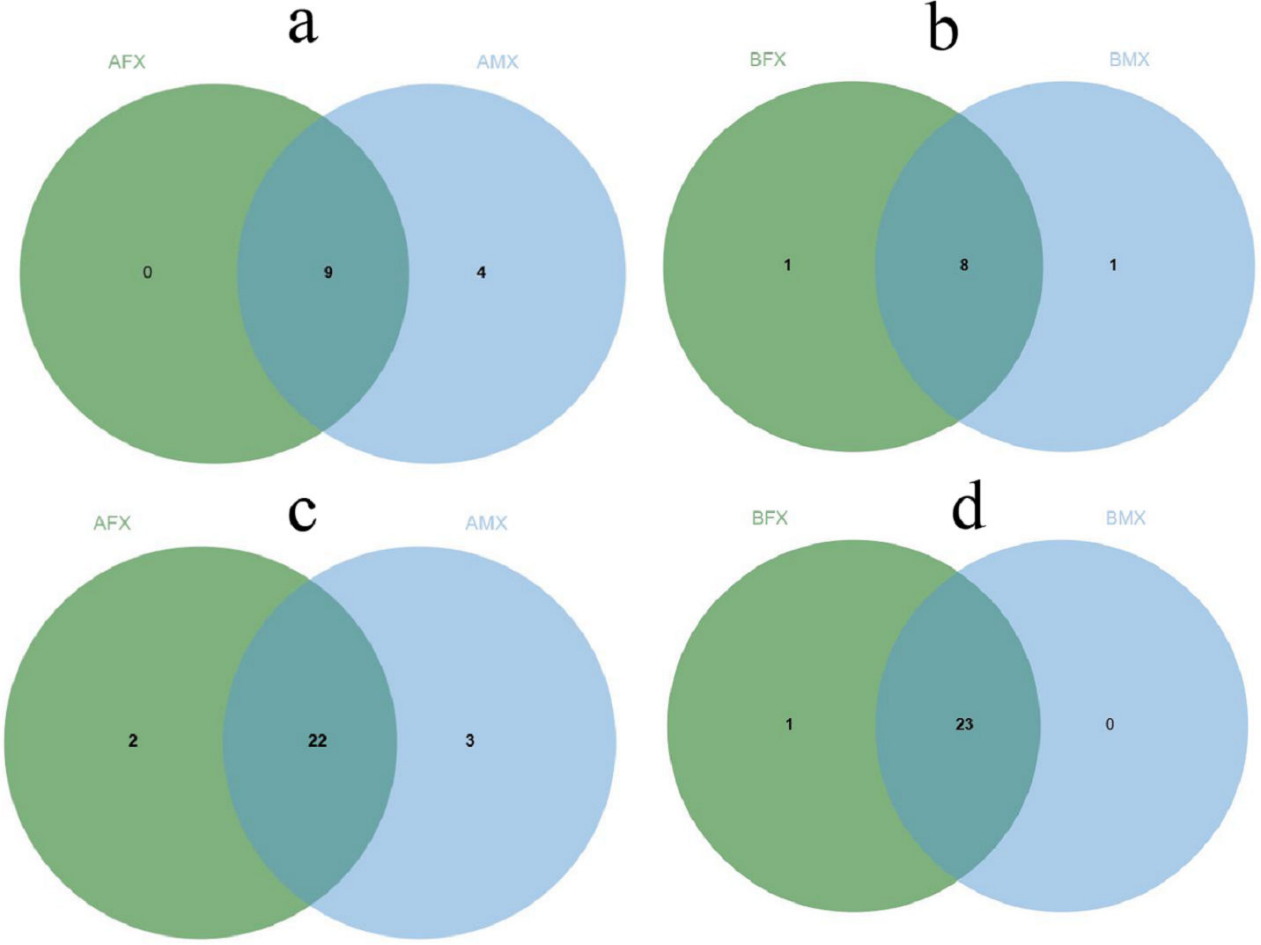
The shared and unique rhizosphere microbial phylum between females and males of the dioecious *H. tibetana* at different habitats. Note: rhizosphere fungal phylum between females and males of the dioecious *H. tibetana* at different habitat A (**a**), Rhizosphere fungal phylum between females and males of the dioecious *H. tibetana* at different habitat B (**b**), Rhizosphere bacterial phylum between females and males of the dioecious *H. tibetana* at different habitat A (**c**), and Rhizosphere bacterial phylum between females and males of the dioecious *H. tibetana* at different habitat B (**d**).

With respect to the fungal phylum level, nine fungal phyla were shared in the AFX and AMX samples. Among them, four fungal phyla were unique in the AMX samples: Basidiobolomycota, Zoopagomycota, Entorrhizomycota, and Monoblepharomycota, while n0 fungal phylum was unique in the AFX samples (Fig. 3a). Eight fungal phyla were shared in the BFX and BMX samples. Among them, two fungal phyla were unique in the BFX and BMX samples, which were Kickxellomycota and Zoopagomycota, respectively (Fig. 3b). In brief, Zoopagomycota was a unique fungal phylum in the males of the dioecious *H. tibetana* (Table S2).

The bacterial OTUs were assigned to 36 phyla and 430 genera in all samples. At the phylum level, Proteobacteria was the dominant bacterial phylum in the AFX, AMX, and BFX samples, with relative abundances of 42.31%, 45.30%, and 51.81%, respectively, while Actinobabacteriota was the dominant bacterial phylum in the BMX sample (41.07%) (Fig. 2c). At the genus level, *Sphingomonas* was the dominant bacterial genus in the AFX, AMX, and BMX samples, with relative abundances of 2.24%, 5.61%, and 3.00%, respectively. *Bradyrhizobium* was the dominant bacterial genus in the BFX sample (3.99%) (Fig. 2d).

With respect to the bacterial phylum level, 22 bacterial phyla were shared in the AFX and AMX samples. Among them, two bacterial phyla were unique in the AFX samples: Sumerlaeota and Dependentiae, and three bacterial phylum were unique in the AMX samples“ Crenarchaeota, Deinococcota, and Hydrogenedentes (Fig. 3c). A total of 23 bacterial phyla were shared in the BFX and BMX samples. Among them, one bacterial phylum were unique in the BFX samples, which was Dependentiae, while no bacterial phylum was unique in the BMX samples (Fig. 3d). In brief, Dependentiae was the unique bacterial phylum in the female of the dioecious *H. tibetana* (Table S2).

**Fig 4:**
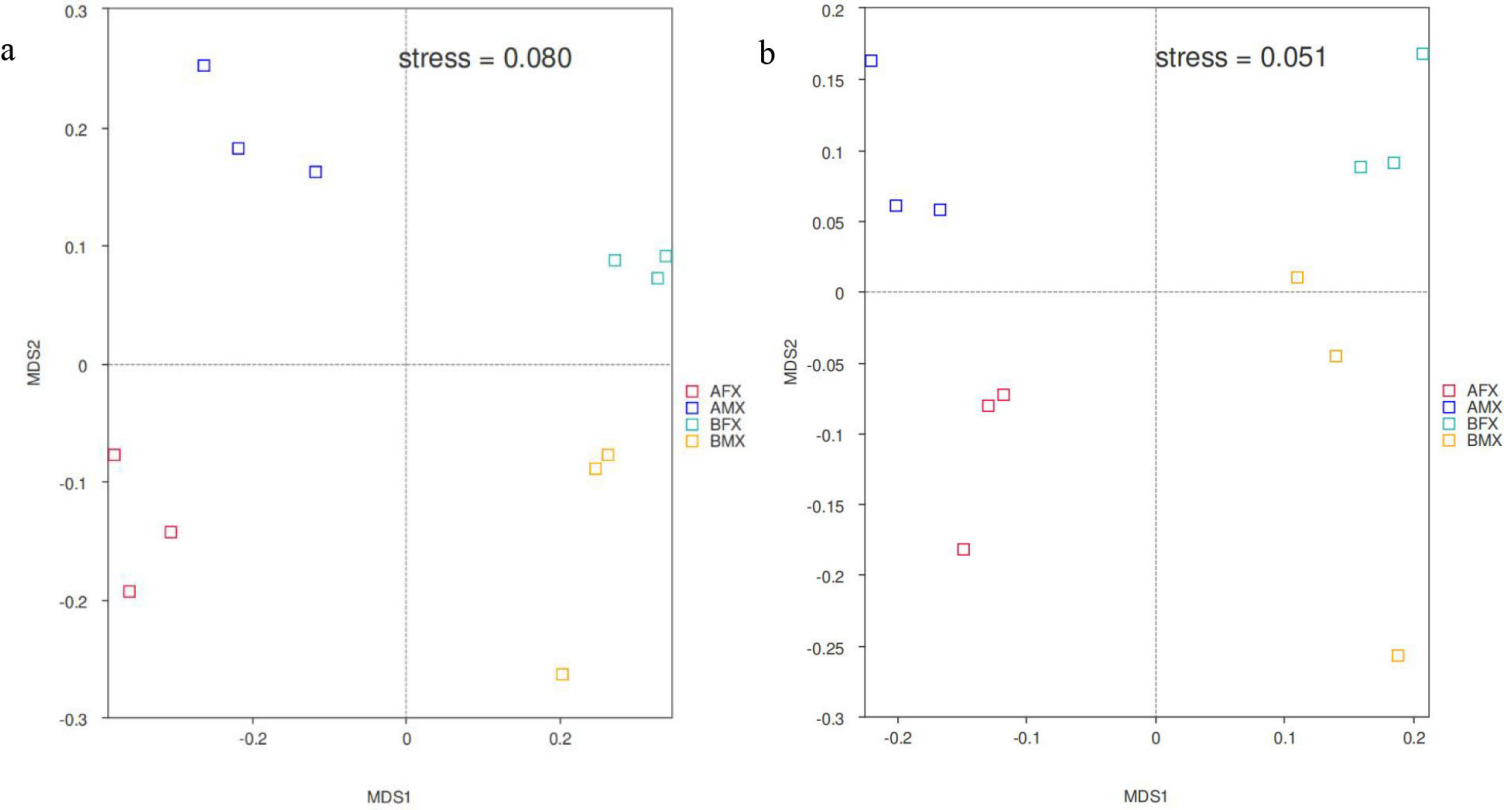
NMDS results of rhizosphere fungal (**a**) and bacterial (**b**) community composition between females and males of the dioecious *H. tibetana* at different habitats.

Each point in the NMDS plot represented a sample, and different colored points indicated different samples (groups). Since NMDS used rank ordering, it can be approximated that the closer (far) the distance between two points, the smaller (greater) the difference in the microbial communities in the two samples. NMDS analysis showed that the fungal and bacterial community composition was generally separated in all samples (Fig. 4). This implied that the community structure of fungi and bacteria has differences between females and males of the dioecious *H. tibetana* at different habitats.

### Biomarkers of females and males of the dioecious *H. tibetana* at different habitats

**Fig 5:**
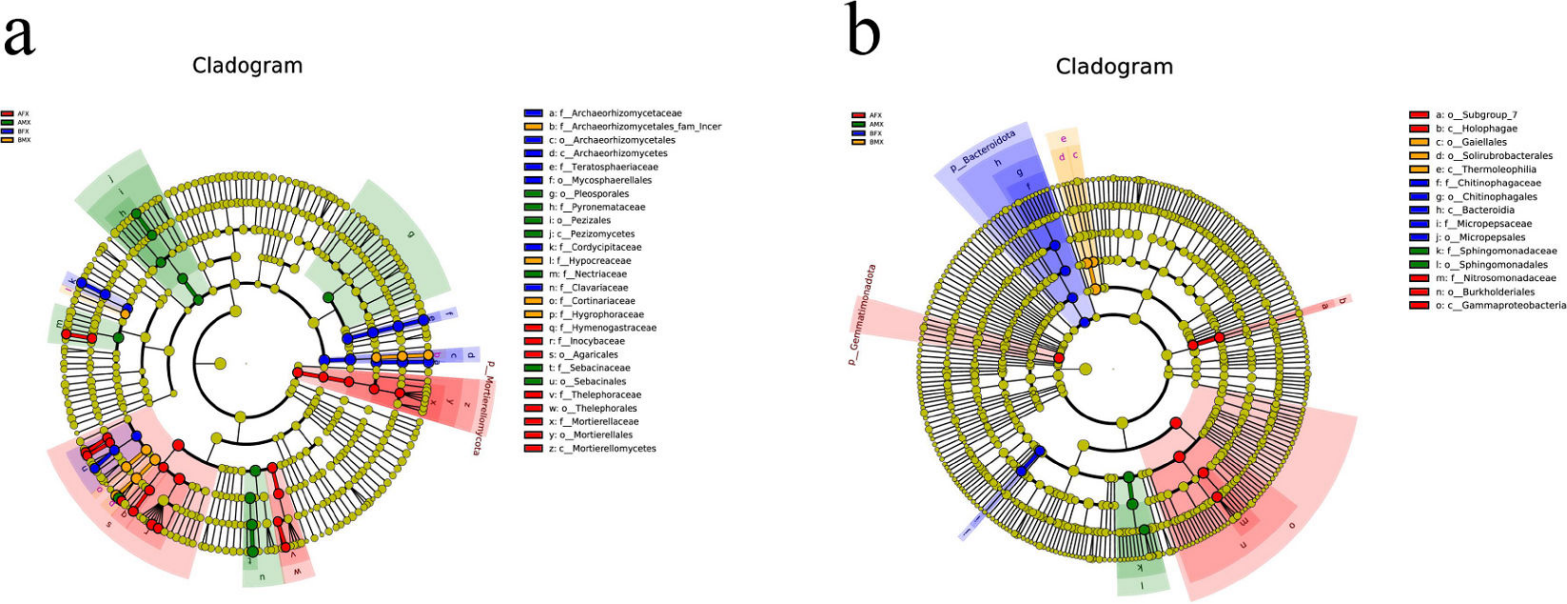
The linear discriminant analysis effect size (LEfSe) analysis between females and males of the dioecious *H. tibetana* at different habitats. Note: Only taxa with LDA >4 and Wilcoxon, *P* < 0.05 are shown. Rhizosphere fungi (**a**) and rhizosphere bacteria (**b**).

The LEfSe analysis revealed differences in the rhizosphere bacterial and fungal communities between females and males of the dioecious *H. tibetana* at different habitats. In the fungal community, there were significant differences at the phylum (Mortierellomycota), the class (Mortierellomycetes), the order (Mortierellales, Thelephorales and Agaricales), the family (Hymenogastraceae, Inocybaceae, Thelephoraceae, and Mortierellaceae), and the genus (*Clavaria*, *Thelonectria*, *Hymenogaster*, *Thelephora*, Clavariaceae_gen_Incertae_sedis, and *Mortierella*) levels in the AFX samples. There were significant differences at the class (Pezizomycetes), the order (Sebacinales, Pleosporales, and Pezizales), the family (Sebacinaceae, Nectriaceae, and Pyronemataceae), and the genus (*Sebacina* and *Geopora*) levels in the AMX samples. There were significant differences at the class (Pezizomycetes), the order (Archaeorhizomycetales and Mycosphaerellales), the family (Archaeorhizomycetaceae, Teratosphaeriaceae, Cordycipitaceae, and Clavariaceae), and the genus (*Archaeorhizomyces*, *Capnocheirides*, *Beauveria,* and *Clavulinopsis*) levels in the BFX samples. There were significant differences at the family (Hygrophoraceae, Cortinariaceae, chaeorhizomycetales_fam_Incertae_sedis, and Hypocreaceae) and the genus (*Hygrocybe*, *Cortinarius,* and *Chaeorhizomycetales*_gen_Incertae_sedis) levels in the BMX samples (Fig. 5a).

In the bacterial community, there were significant differences at the phylum (Gemmatimonadota), the class (Gammaproteobacteria and Holophagae), the order (Burkholderiales and Subgroup_7), the family (Nitrosomonadaceae), and the genus (*Ellin*6067) levels in the AFX samples. There were significant differences at the order (Sphingomonadales), the family (Sphingomonadaceae), and the genus (*Sphingomonas*) levels in the AMX samples. There were significant differences at the phylum (Bacteroidota), the class (Bacteroidia), the order (Chitinophagales and Micropepsales), and the family (Chitinophagaceae and Micropepsaceae) levels in the BFX samples. There were significant differences at the class (Thermoleophilia) and order (Solirubrobacterales and Gaiellales) levels in the BMX samples (Fig. 5b).

### Co-occurrence network analysis of rhizosphere fungal and bacterial community

To explore whether changes in microbial community assemblies were accompanied by changes in microbial interactions, we performed co-occurrence network analysis and estimated the topological properties to uncover the complexity of potential associations and connections between females and males of the dioecious *H. tibetana* at different habitats.

**Fig 6:**
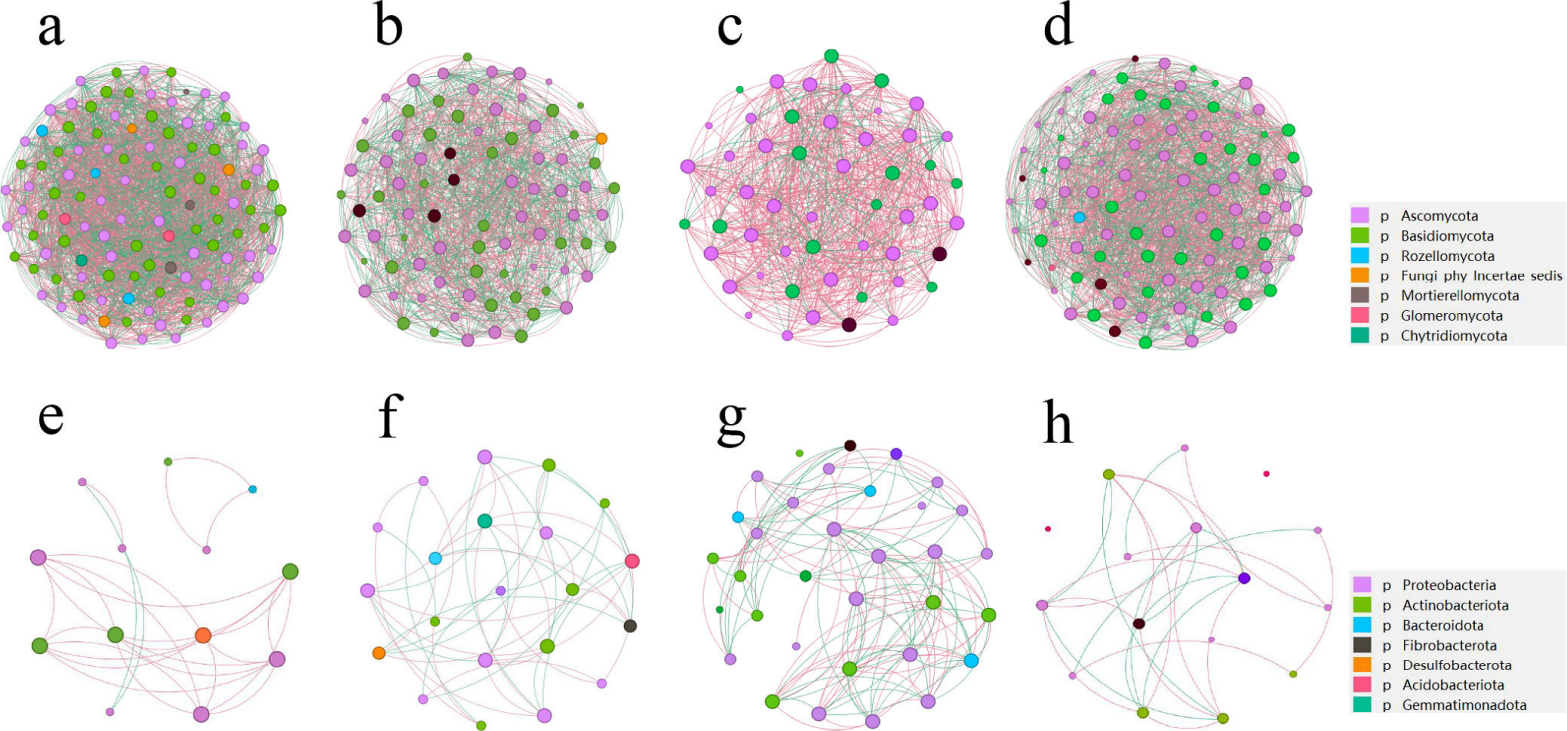
Co-occurrence network analysis between females and males of the dioecious *H. tibetana* at different habitats. Note: Co-occurrence network diagram of female rhizosphere fungi under habitat A (**a**), Co-occurrence network diagram of male rhizosphere fungi under habitat A (**b**), Co-occurrence network diagram of female rhizosphere fungi under habitat B (**c**), Co-occurrence network diagram of male rhizosphere fungi under habitat B (**d**), Co-occurrence network diagram of female rhizosphere bacteria under habitat A (**e**), Co-occurrence network diagram of male rhizosphere bacteria under habitat A (**f**), Co-occurrence network diagram of female rhizosphere bacteria under habitat B (**g**), Co-occurrence network diagram of male rhizosphere bacteria under habitat B (**h**).

Co-occurrence network analysis revealed that rhizosphere fungal and bacterial co-occurrence network connectivity and complexity showed differences between females and males of the dioecious *H. tibetana* at different habitats (Fig. 6; Table S3). In the fungal community, total number of nodes, links, positive edges, negative edges, relative modularity, and map density of AFX samples were higher than those of AMX samples (Fig. 6a and b; Table S3), whereas the total number of nodes, links, positive edges, negative edges, relative modularity, and map density of BFX samples was lower than that of BMX samples (Fig. 6c and d; Table S3). In the bacterial community, the total number of nodes, links, positive edges, negative edges, relative modularity, and map density of AFX samples was lower than that of AMX samples (Fig. 6e and f; Table S3), whereas the total number of nodes, links, positive edges, negative edges, relative modularity, and map density of BFX samples was higher than that of BMX samples (Fig. 6g and h; Table S3).

### Spearman correlation analysis between rhizosphere microbes and soil physicochemical properties

**Fig 7:**
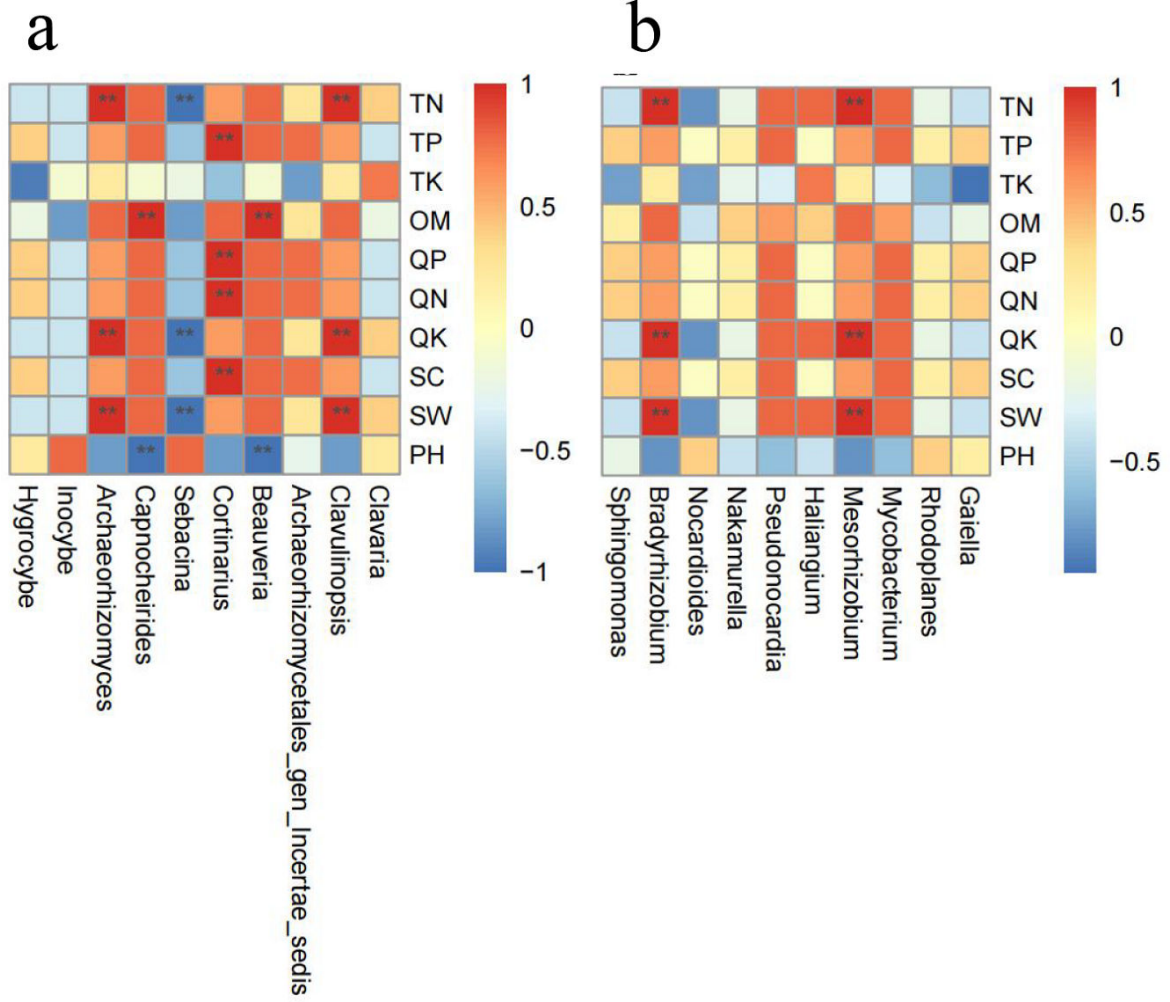
Correlation analysis between rhizosphere microbes and soil physicochemical properties. Note: Spearman correlation analysis between the genus of rhizosphere fungi and soil physicochemical properties (**a**) . Spearman correlation analysis between the genus of rhizosphere bacteria and soil physicochemical properties (**b**). “*” represents that the differences are significant at *P* < 0.05, “**” represents that the differences are significant at *P* < 0.01 (Wilcoxon).

Correlation analysis was performed based on the relative abundance of ten dominant fungal and bacterial genera with the soil physicochemical properties. The Spearman correlation analysis showed that *Clavulinopsis* and *Archaeorhizomyces* were significantly positively correlated with TN, QK, and SW (*P* < 0.01), *Beauveria* and *Capnocheirides* were significantly positively correlated with OM. *Cortinarins* was significantly positively correlated with TP, QP, QN, and SC (*P* < 0.01). *Sebacina* was significantly negatively correlated with TN, QK, and SW. *Beauveria* and *Capnocheirides* had a significant negative correlation with pH (*P* < 0.01) (Fig. 7a).

*Mesorhizobium* and *Bradyrhizobium* were significantly positively correlated with TN, QK, and SW (*P* < 0.01) (Fig. 5b).

### FUNGuild and PICRUSt functional prediction analysis

**Fig 8:**
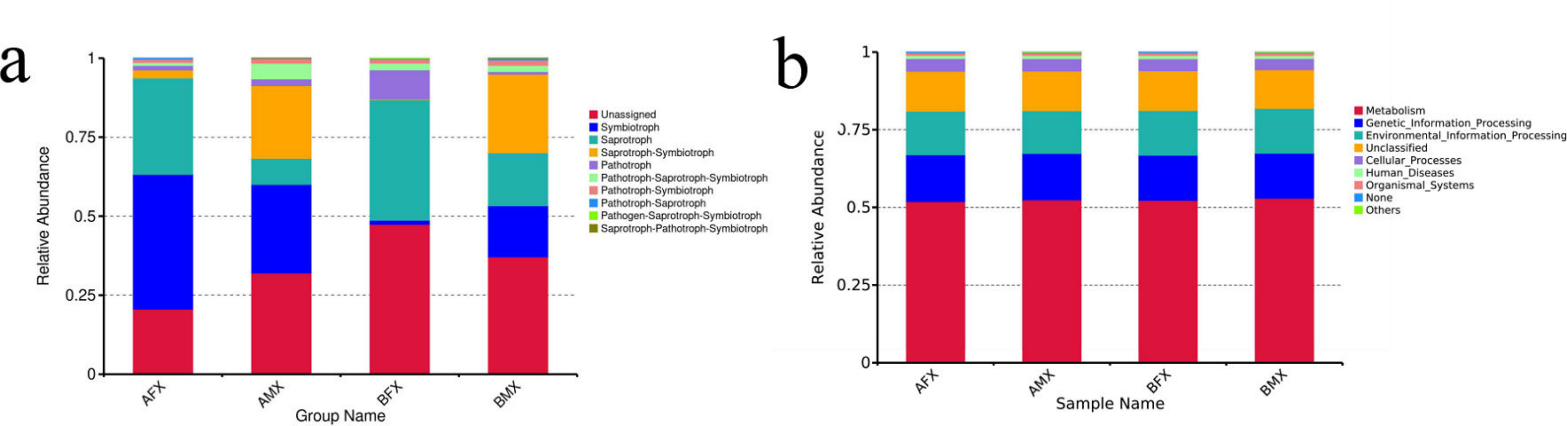
Relative abundance of the predicted trophic mode of fungi (**a**) . Relative abundance of predicted KEGG Orthologs functional profiles (KEGG level 1) of bacteria (**b**).

FUNGuild was used to predict the nutritional and functional groups of the fungal communities with different samples. The results of rhizosphere fungal function prediction showed that nine trophic mode groups could be classified: Saprotroph–Pathotroph–Symbiotroph, Pathogen–Saprotroph–Symbiotroph, Pathotroph–Saprotroph, Pathotroph–Symbiotroph, Pathotroph–Saprotroph–Symbiotroph, Pathotroph, Saprotroph–Symbiotroph, Saprotroph, and Symbiotroph. The Symbiotroph was the preponderant trophic mode in the AFX and AMX samples, accounting for 43.63% and 28.01%, respectively. The Saprotroph was the preponderant trophic mode in the BFX samples (38.10%), Saprotroph–Symbiotroph was the preponderant trophic mode in the BMX samples (23.66%). (Fig. 8a).

To study the bacterial function, we used PICRUSt to perform bacterial function prediction analysis. Through a comparison with the Kyoto Encyclopedia of Genes and Genomes (KEGG) database, we have obtained six primary functional categories of metabolic pathways (primary functional level): metabolism, gene and environmental information processing, cellular processes, organ systems, and human diseases. The metabolism was the main function in all samples, accounting for 51.88%–52.42% (Fig. 8b).

## DISCUSSION

The differences between dioecious plants has long been the focus of research by plant ecologists. Differences of morphology, resource allocation, reproductive allocation, stress adaptation, and nutrient availability have been extensively studied between male and female dioecious plants (25–27). Previous studies showed that there were significant differences in the morphology, chlorophyll content, stomatal distribution, leaf senescence rate, and physiological metabolism between male and female plants of *H. tibetana* (28). Although the above studies proved the differences in the morphology and physiological metabolism between females and males of the dioecious *H. tibetana*, the differences in the composition of rhizosphere microbes between females and males of the dioecious *H. tibetana* are not clear. In this study, we aimed to explore the differences in rhizosphere microbial communities between females and males of the dioecious *H. tibetana* at different habitats and attempted to better understand sex-specific interactions, mechanisms, and functions underground. The results indicated that the diversity and composition showed differences: Proteobacteria was the dominant phylum in all samples. Proteobacteria contains a large number of oligotrophic microbial functional groups, and a considerable part of the groups have the ability of nitrogen fixation, metabolic activities using nutrients generated by the decomposition of various organic substances, and strong adaptability to the environment (29), which may be an important reason why Proteobacteria is the main dominant phylum in all samples. Ascomycota and Basidiomycota were dominant phyla in all samples; most fungi can enable the fungal species to resist abiotic stress factors, such as temperature stress and UV irradiation stress, and hence have been observed as the most abundant phyla of soil in many regions (3). Some fungi in the phylum Ascomycota played an vital role in the survival of plants under harsh conditions (30). Meanwhile, Basidiomycota was also identified as a dominant phylum. It has been reported that the species of these two phyla form symbiotic associations with plants, which can further promote the growth and development of the host plants (31). These results are similar to those of previous studies (30). Interestingly, we found that there were unique phyla of rhizosphere microbea between females and males of the dioecious *H. tibetana* at different habitata. Zoopagomycota was a unique phylum of rhizosphere fungi in the males of the dioecious *H. tibetana*, while Dependentiae was a unique phylum of rhizosphere bacteria in the females of the dioecious *H. tibetana*. It indicated that there may be some relationship between unique rhizosphere microbes and plant sex, but the relationship needs to be explored further. Moreover, we used LEfSe analysis to find out the differences in rhizosphere microbes at different levels, which can be used as potential biomarkers, and those potential biomarkers were different between females and males of the dioecious *H. tibetana* at different habitats. In further studies, we may use these potential biomarkers to distinguish between females and males of the dioecious *H. tibetana*. In addition, co-occurrence network analysis revealed that rhizosphere microbial co-occurrence network connectivity and complexity had differences between females and males of the dioecious *H. tibetana* at different habitats, and the rhizosphere fungal co-occurrence network of *H. tibetana* was more complex. The results were similar to the reports of Guo *et al*. (32). Variations in the constituting microbial taxa have a significant influence on the assembly of microbial communities, and this effect is suggested to be independent from external environmental factors (33). Many studies also reported that microbial community composition had complex interactions with environmental variables (34). The richness and community composition of microbes could be severely affected by environmental factors (35). In order to clarify the impact of environmental factors on differences of rhizosphere microbes between females and males of the dioecious *H. tibetana*, Spearman’s correlation analysis between rhizosphere microbe and soil physicochemical properties was tested, we found that there was a significantly positive correlation between some soil physicochemical properties and dominant genera of rhizosphere microbes. A previous study has demonstrated the important effects of plant hosts on the rhizosphere microbes via deterministic selection during the growth and development of plants (36). In this study, female and male plants of *H. tibetana* exerted gender-specific selection pressures or provided specific habitats to shape distinct rhizosphere microbial communities. The findings suggest the significance of considering sex-specific rhizosphere microbial assembly in ecological restoration processes.

FUNGuild has been employed for the examination of fungal function, which corresponds to the distinct functional categorization of fungi. Recently, there has been extensive utilization of it for analysis of fungal populations (37). This study found that there were differences in the preponderant trophic mode of rhizosphere fungi between females and males of the dioecious *H. tibetana* at different habitats, which indicated the trophic mode of rhizosphere fungi was influenced by environmental factors and sex. PICRUSt analysis can accurately predict metabolic activities of bacterial communitie (38). In this study, we use high-throughput sequencing data to forecast the functions of rhizosphere bacteria through PICRUSt. The results showed that the metabolism was the dominant function in females and males of the dioecious *H. tibetana* samples, which the functions of rhizosphere bacteria was not influenced by environmental factors and sex.

### Conclusions

Overall, we provided novel evidence that there were differences in rhizosphere microbial communities between females and males of the dioecious *H. tibetana* at different habitats, and there existed unique phyla of rhizosphere microbes and potential biomarkers of rhizosphere microbes at different levels between females and males of the dioecious *H. tibetana*. Rhizosphere fungi were significantly positively correlated with soil physicochemical properties. Our results provided important knowledge for the impact of sexual dimorphism in the host plant on the assembly of rhizosphere microbes and interaction between dioecious plants and microbes. It also contributes to lay a theoretical foundation for revealing the environmental adaptation strategies of dioecious plants from the perspective of microbes.

## Data Availability

All raw sequencing data have been submitted to the NCBI Sequence Read Archive (SRA) database under the accession number (PRJNA1108968).

## SUPPLEMENTAL MATERIAL

Figure S1 spectrum.01670-24-s0001.tif Fig. S1: Sample collection sites.

Tables S1 to S3 spectrum.01670-24-s0002.docx Table S1: Alpha diversity of rhizosphere fungi and bacteria. Table S2: The unique rhizosphere microbial phylum. Table S3: Analysis of rhizosphere fungal and bacterial co-occurrence networks.

ASM does not own the copyrights to Supplemental Material that may be linked to, or accessed through, an article. The authors have granted ASM a non-exclusive, world-wide license to publish the Supplemental Material files. Please contact the corresponding author directly for reuse.
