# Supplementary figures and images for: Divergence of rhizosphere microbial communities between females and males of the dioecious Hippophae tibetana at different habitats
Source: Microbiol Spectr. 2024 Sep 11;12(10):e01670-24. doi: 10.1128/spectrum.01670-24 (PMC11448439; doi:10.1128/spectrum.01670-24)

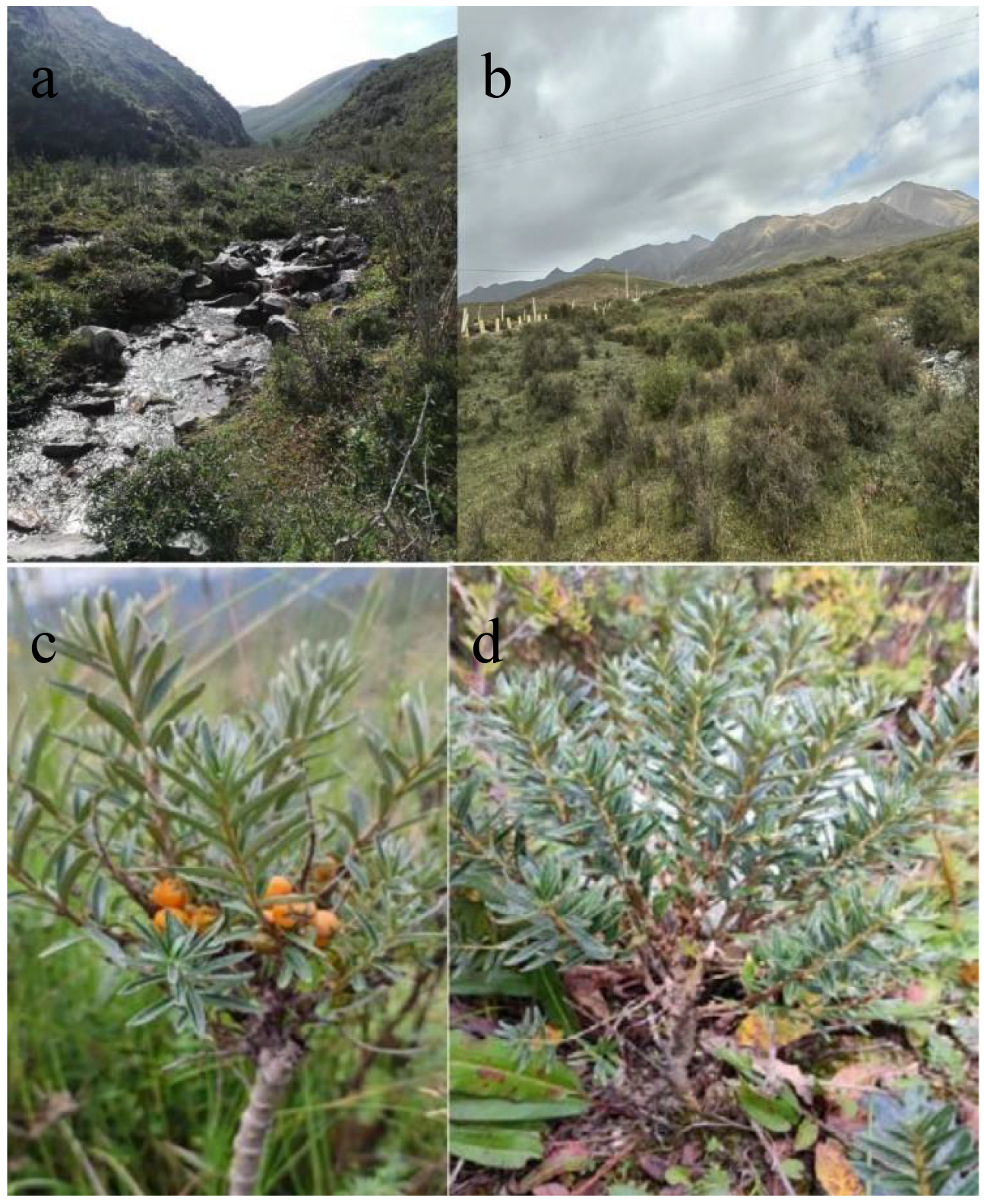

Supplement: Figure S1 — Fig. S1: Sample collection sites. [file spectrum.01670-24-s0001.tif]
