# Supplementary material for: Divergence of rhizosphere microbial communities between females and males of the dioecious Hippophae tibetana at different habitats
Source: Microbiol Spectr. 2024 Sep 11;12(10):e01670-24. doi: 10.1128/spectrum.01670-24 (PMC11448439; doi:10.1128/spectrum.01670-24)
Supplement: Tables S1 to S3 — Table S1: Alpha diversity of rhizosphere fungi and bacteria. Table S2: The unique rhizosphere microbial phylum. Table S3: Analysis of rhizosphere fungal and bacterial co-occurrence networks. [file spectrum.01670-24-s0002.docx]

Table S1 Alpha diversity of rhizosphere fungi and bacteria between females and males of the dioecious *H. tibetana* at different habitat

|  | Fungi | | | | Bacteria | | | |
| --- | --- | --- | --- | --- | --- | --- | --- | --- |
| Sample | Effective Tags | chao1 | Shannon | Coverage | Effective Tags | chao1 | Shannon | Coverage |
| AFX | 96,820 | 551.06±25.07a | 5.97±0.30a | 0.999 | 60,642 | 1774.28±74.08c | 9.81±0.06b | 0.998 |
| AMX | 97,396 | 490.83±33.88b | 4.81±0.10b | 0.998 | 82,203 | 2117.92±51.57ab | 10.00±0.09ab | 0.999 |
| BFX | 94,992 | 465.08±40.66b | 5.66±0.45ab | 0.999 | 99,254 | 2349.04±149.21a | 10.08±0.04a | 0.997 |
| BMX | 103,806 | 487.36±31.06b | 5.02±0.30ab | 0.999 | 66,077 | 1924.73±36.75bc | 9.85±0.04b | 0.998 |

Note：Different letters above the bars indicate the differences are significant at *P* < 0.05.

Table S2 The unique rhizosphere microbial phylum

|  | Sample | Unique rhizosphere microbial phylum | | | |
| --- | --- | --- | --- | --- | --- |
| Fungi | AFX |  |  |  |  |
|  | AMX | Basidiobolomycota | Zoopagomycota | Entorrhizomycota | Monoblepharomycota |
|  | BFX | Kickxellomycota |  |  |  |
|  | BMX | Zoopagomycota |  |  |  |
| Bacteria | AFX | Sumerlaeota | Dependentiae |  |  |
|  | AMX | Crenarchaeota | Deinococcota | Hydrogenedentes |  |
|  | BFX | Dependentiae |  |  |  |
|  | BMX |  |  |  |  |

Table S3 Analysis of rhizosphere fungal and bacterial co-occurrence networks between females and males of the dioecious *H. tibetana* at different habitat

|  | Sample | Total number of nodes | Total number of links | Positive edges | Negative edges | Relative modularity | Map density |
| --- | --- | --- | --- | --- | --- | --- | --- |
|  | AFX | 113 | 2074 | 1118 | 955 | 0.614 | 0.328 |
| Fungi | AMX | 83 | 1067 | 545 | 521 | 0.556 | 0.314 |
|  | BFX | 52 | 581 | 495 | 85 | 0.384 | 0.338 |
|  | BMX | 97 | 1574 | 917 | 656 | 0.627 | 0.438 |
|  | AFX | 13 | 27 | 24 | 3 | 0.370 | 0.229 |
| Bacteria | AMX | 21 | 48 | 27 | 21 | 0.680 | 0.346 |
|  | BFX | 31 | 135 | 69 | 65 | 0.580 | 0.370 |
|  | BMX | 13 | 27 | 16 | 11 | 0.225 | 0.256 |
